# Intervention Component Analysis (ICA): a pragmatic approach for identifying the critical features of complex interventions

**DOI:** 10.1186/s13643-015-0126-z

**Published:** 2015-10-29

**Authors:** Katy Sutcliffe, James Thomas, Gillian Stokes, Kate Hinds, Mukdarut Bangpan

**Affiliations:** EPPI-Centre, Social Science Research Unit, Institute of Education, University College London (UCL), 18 Woburn Square, London, WC1H 0NS UK

**Keywords:** Systematic reviews, Evidence synthesis, Complex interventions, Knowledge translation, Paediatrics, Medication error, Electronic prescribing

## Abstract

**Background:**

In order to enable replication of effective complex interventions, systematic reviews need to provide evidence about their critical features and clear procedural details for their implementation. Currently, few systematic reviews provide sufficient guidance of this sort.

**Methods:**

Through a worked example, this paper reports on a methodological approach, Intervention Component Analysis (ICA), specifically developed to bridge the gap between evidence of effectiveness and practical implementation of interventions. By (a) using an inductive approach to explore the nature of intervention features and (b) making use of trialists’ informally reported experience-based evidence, the approach is designed to overcome the deficiencies of poor reporting which often hinders knowledge translation work whilst also avoiding the need to invest significant amounts of time and resources in following up details with authors.

**Results:**

A key strength of the approach is its ability to reveal hidden or overlooked intervention features and barriers and facilitators only identified in practical application of interventions. It is thus especially useful where hypothesised mechanisms in an existing programme theory have failed. A further benefit of the approach is its ability to identify potentially new configurations of components that have not yet been evaluated.

**Conclusions:**

ICA is a formal and rigorous yet relatively streamlined approach to identify key intervention content and implementation processes. ICA addresses a critical need for knowledge translation around complex interventions to support policy decisions and evidence implementation.

## Background

### Enhancing the utility of evidence for policy decisions

Whilst the body of systematic reviews examining the effectiveness of interventions is burgeoning [1], currently few systematic reviews provide sufficient guidance to enable practical application of the evidence synthesised [2]. To enable replication of effective complex health service interventions, systematic reviews need to provide evidence about the critical features of interventions and clear procedural details for their implementation.

The current lack of such information has been attributed to a number of factors. First, some argue that to date, there has been a lack of awareness among systematic reviewers of the information needs of users of systematic reviews [2]. A second contributor to the problem is the often substandard reporting of intervention details in primary studies [3, 4]. A third reason is the complexity of many non-pharmacological interventions [5, 6] which in turn undermines the ability of established statistical approaches to distinguish the impact of different intervention features on outcomes. This paper reports on a methodological approach, Intervention Component Analysis (ICA), specifically developed to overcome these challenges and to bridge the gap between evidence of effectiveness and practical implementation of interventions.

### Understanding the needs of review users

At the EPPI-Centre, we have been sensitised to the information needs of decision-makers through our long-standing programme of work with the Department of Health, England, to undertake systematic reviews to inform policy. One feature that distinguishes many of these reviews to inform policy development (and implementation) is that they tend to be broader than systematic reviews carried out to inform decisions in clinical situations. Rather than beginning with the standard PICO framework, which defines the Population, Intervention, Comparator and Outcome of interest, the policymaker often begins with an Outcome (or outcomes) of interest and seeks to identify a range of approaches which might improve this/those outcome/s (such as ‘healthy eating’, ‘physical activity’, etc.). Populations tend to be fairly broad: ‘adults’, ‘children’ or ‘young people’, rather than very narrow age ranges or those with specific pre-existing conditions. Such reviews are less concerned with identifying a single pooled effect size estimate, as may be the case in clinical reviews, than with identifying intervention approaches which may be useful in particular situations or with particular population (sub)groups. Thus, as well as addressing questions of effectiveness (‘what works?’), reviews to inform policy also need to answer the question ‘what works, for whom, in what situation?’. In addition, reviews for policy also utilise a much wider range of evidence than early clinical systematic reviews with their exclusive focus upon randomised controlled trials (RCTs) [7, 8]. Some policy questions go beyond effectiveness (e.g. ‘is there a problem?’, ‘what can we do about..?’, ‘what are people’s experiences of..?’, and ‘can a particular intervention be implemented in this context?’); thus, different types of research are required in addition to trials, including epidemiological research (such as surveys and cohort studies) and qualitative studies. The EPPI-Centre works to develop systematic review methods to accommodate the broad range of policy-relevant questions and the concomitant range of research. The ICA approach described in this paper is one such development.

### Addressing the problem of poor quality intervention descriptions

Despite the fact that the 2010 Consolidated Standards of Reporting Trials (CONSORT) statement recommends that authors should report interventions with ‘sufficient details to allow replication’ [9], evidence indicates that substandard reporting of intervention details is widespread [3, 4]. Thus, even if reviewers are sufficiently sensitised to decision-makers’ implementation needs, accessing sufficient information from primary studies will remain a significant challenge.

To remedy this situation, the ‘TIDieR’ Checklist has been designed to supplement the CONSORT statement with a view to improving the reporting of interventions; the authors are also mindful of the implications for systematic reviews and review users [10]. Nevertheless, given the information needs of review users, and pending the impact of efforts such as TIDieR to ensure improvements in reporting, reviewers need to find ways to overcome the current problem of ‘remarkably poor’ reporting of intervention details [10]. One suggested approach is to contact trial authors for additional details [3, 11]. However, this approach has been found to require ‘considerable extra work’ compared to a standard review, which may not always be feasible [2]. In some cases, it has also produced limited results: in their study of RCTs of non-pharmaceutical interventions, Hoffmann and colleagues found that only 39 % of published trials provided adequate description of interventions, and the rate of adequate information increased to just 59 % following attempts to contact authors [3].

### The challenge of reporting complex interventions

Interventions that are carried out in a social context are prone to poor reporting, due to their complexity. The term ‘complex intervention’ is in common use, and there is the Medical Research Council guidance on how to develop and evaluate such interventions [12]. This guidance defines complexity in terms of the following:

- Number of interactions between components
- Number and difficulty of behaviours required by those delivering or receiving the intervention
- Number of groups or organisational levels targeted by the intervention
- Number and variability of outcomes
- Degree of flexibility or tailoring of the intervention permitted (p. 7)

However, whilst it may be difficult to identify the ‘active ingredients’ of an intervention with the above characteristics, some of these issues may be considered as merely ‘complicated’; in an alternative conceptualisation, complex interventions are characterised by ‘recursive causality (with reinforcing loops), disproportionate relationships (where at critical levels, a small change can make a big difference—a ‘tipping point’) and emergent outcomes’ [13].

The distinction between an intervention and the context in which it is implemented is often less clear in the case of complex interventions meaning that mechanisms of action or causal pathways are often obscured [6]. Wells and colleagues argue that ‘There is potential for the cause to be: the intervention itself, elements of the healthcare context within which the intervention is being delivered, elements of the research process that are introduced to that setting (for example, presence of researchers and their operations), or a combination of all three’ [6]. Multiple potential influences will necessarily increase the challenge of providing an accurate and comprehensive description in trial reports. Moreover, it is posited that researchers may be reticent to recognise or report the influence of contextual factors on the delivery or impact of interventions since an inherent aim of randomised controlled trials is to control and standardise the interventions under study [6].

### The challenge of identifying critical components in complex interventions

Statistical approaches for exploring variation in outcomes have a long history of use in systematic reviews, including methods such as meta-regression, subgroup analysis, network meta-analysis and various other moderator analyses [14, 15]. These statistical methods, which partition and attempt to explain between-study variance in different ways, are a powerful way of identifying which intervention characteristics might be necessary and/or sufficient, individually or in combination, to bring about a given outcome. Unfortunately, they all share one significant weakness in terms of their utility: they depend on intervention *replication* in order to operate effectively. In situations in which each intervention in a review may differ from another (in sometimes subtle ways), they have less traction; and in evaluations of social interventions, there are very few genuine replications. This means that systematic review datasets often lack the necessary numbers of studies that might allow them to explore meaningful numbers of mediators and moderators of intervention effect. They are therefore often unable to identify which intervention characteristics should be selected by decision-makers in different situations. When faced with unexplainable heterogeneity, most reviewers resort to a ‘narrative’ synthesis, in which the results of studies are compared to one another thematically [16].

Moreover, when considered alongside the brief discussion earlier about the nature of complicated and complex interventions, and the need to identify ‘active ingredients’ for decision-makers, correlation-based methods have some serious weaknesses, which limit their suitability for use in these situations; most importantly, typically they do not allow for the possibility of multiple causal configurations. Correlation tests are symmetric in nature, which means that when the strength of a given relationship is tested, part of the test requires the absence of a non-relationship—which may not make sense conceptually, and is certainly a problem in terms of detecting multiple causal pathways. For example, in an analysis which is examining whether training intervention providers results in better outcomes, a correlational analysis will require that training intervention providers is associated with good outcomes *and* that the absence of training providers is associated with poorer outcomes. However, if there are multiple routes through to effectiveness, it may be that some (or all) of the interventions where training did not occur had good reasons for this (e.g. they had recruited more experienced providers) but this would not be picked up in the analysis, and the importance of training in the interventions which did train providers would be lost. There are, of course, refinements to simple correlational tests which test for interactions between variables (e.g. training and/or experience of providers), but they tend to require more data (i.e. more replications of sufficiently similar interventions) to operate.

As such, the ability of conventional statistical approaches to detect key intervention features is questionable in the arena of complex interventions. However, if policymakers and practitioners are to base their decisions on the findings of systematic reviews, they still require information to guide the selection and prescription of a specific approach. Whilst it may not be possible to *test* hypotheses reliably using the statistical approaches described above, the development and *articulation* of hypotheses about critical intervention features may nevertheless provide further insight for decision-makers.

### Challenges to developing and articulating theories of intervention mechanisms

Developing theory or presenting existing theoretical models as part of a review has been recommended as one approach to address the ‘missing link’ with regard to translation of evidence for decision-makers [2, 17]. One of the tools which is gaining increasing popularity is the use of *programme theory* or *logic models* to detail the causal pathway(s) through which an intervention is intended to have its effect [18]. Often depicted in diagrammatic form, logic models can illustrate connections between outcomes and what influences them, describe mediators and moderators and specify intermediate outcomes and possible harms [19].

However, whilst articulation of the theoretical basis of interventions can help with knowledge translation, there are a number of potential weaknesses to this approach [20]. First, the programme theories may not correctly identify and understand all relevant mechanisms; evidence suggests that important mechanisms are often missed and that identified mechanisms sometimes have ‘surprising side effects’ [20]. Second, the programme theory is only helpful if the intervention is implemented correctly, such that the theoretical mechanisms remain intact.

Moreover, whilst theory can help to explain how or why a particular intervention or component *might* work, opportunities to build on the experience of actually implementing and delivering specific interventions are still missed if a priori theory is the sole focus of analysis. Experiential knowledge can uncover potentially neglected aspects of interventions which are actually important for their effectiveness. As Penelope Hawe notes in her discussion of improvement science, consideration of valuable experiential knowledge comes ‘from the hands of practitioners/implementers’ and that ‘failure to acknowledge this may blind us to the very mechanisms we seek to understand’ [21] Building on experience can be done by integrating evidence on the effectiveness of complex interventions with other types of research evidence [22], for example, qualitative studies which explore the views and experiences of intervention recipients or providers [23, 24]. But, unless the experiential data is drawn from process evaluations conducted alongside effectiveness trials, we are not able to build on lessons learned about key ingredients during the *practical application* of effective interventions and in particular in situations where the programme theory is not supported by the evidence. Unfortunately, learning from practical application is often hampered by a lack of high-quality process evaluations [25, 26].

### The context in which Intervention Component Analysis was developed

The ICA approach, described through a worked example below, was developed in a review examining the effectiveness of interventions to reduce medication errors in children. We focus here on one type of intervention examined in that review: paediatric electronic prescribing. Often referred to as Computerised Prescription Order Entry (CPOE), electronic prescribing involves entering and processing prescription orders via computer, as opposed to traditional paper-based systems.

The review commissioners specifically requested an exploration of implementation issues as part of the review, and we were faced with many of the factors and challenges described above. Electronic prescribing is a complex intervention, owing to the potential for variance in the features of computer programmes as well as the context within which CPOE is used. For CPOE, the programme theory is well established: it is hypothesised that medication errors are reduced as electronic prescribing enables access to clinical decision support at the point of care and ensures that orders are legible and complete; yet, we found a large amount of variance in outcomes among the included studies. We were also hampered by a lack of detail about the nature of electronic prescribing; the quality of many intervention descriptions was suboptimal, and very few trials conducted process evaluations. Producing the review according to the policy timetable precluded the time-consuming options of seeking additional qualitative studies or conducting multiple rounds of author survey.

The review, therefore, needed to adopt a novel approach to assist decision-makers in understanding the critical components of paediatric electronic prescribing packages.

### What is Intervention Component Analysis and how does it overcome the challenges of identifying critical components of complex interventions?

In essence, the ICA approach aims to identify what an effective intervention ‘looks like‘. It is suitable for situations where we have a group of similar interventions—aiming to impact on the same outcome—but which differ from one another in small ways, and we do not know which are important in terms of impacting on the outcome. However, it is particularly appropriate in situations where existing programme theory has been unable to explain variance in outcomes *and* where there is suboptimal information about the interventions under scrutiny (i.e. low-quality intervention descriptions and a lack of process evaluations). Two key approaches are employed in the analysis of trial reports to overcome these challenges. First, qualitative data analysis techniques are employed to develop an inductively derived understanding of the nature of interventions; the inductive approach aims to ensure meticulous analysis of intervention descriptions and to facilitate comparison across the set of studies. Second, to augment the understanding of the intervention, informally reported evidence about the experience of developing and implementing the intervention is captured from the discussion sections of trial reports. See Table 1 for a summary of ICA.

**Table 1:** Overview of the ICA approach

Aims to answer…	○ How do interventions differ from one another?
	○ Which of these differences in characteristics appears to be important?
	○ What would an ideal version of the intervention ‘look like’?
Assumes that…	○ Characteristics that are present in all/many effective interventions are worthy of attention
	○ Characteristics that may be present in one/small numbers of intervention(s) may be less important
Addresses the challenge of poor quality intervention descriptions by…	○ Using an inductive approach to explore the nature of intervention features
	○ Making use of trialists’ informally reported experience-based evidence

## Methods

The method involves two stages. The first seeks to identify how interventions differ from one another; the second then seeks to identify which of these differences in intervention characteristics appear(s) to be important. The first stage, understanding differences between interventions, contained two distinct and parallel processes: the ‘effectiveness synthesis’ carried out a standard ‘narrative’ analysis which sought to identify any clinically significant differences in the outcomes reported by the evaluations; and in parallel to this, another team of researchers carried out the first stage of the ICA—understanding the characteristics of included interventions in detail.

**Fig. 1:**
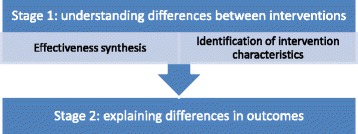
Structure and sequencing of analyses

The results of the two pieces of analysis—the success/failure of individual studies, and the detailed classifications of individual study characteristics—were then combined in the final stage of the ICA, which sought to understand variation in outcomes through mapping them against the intervention characteristics. This analytical structure is summarised in Fig. 1.

### Stage one: understanding how interventions differ from one another

In the first stage, we gathered as much evidence as possible about the nature of the electronic prescribing intervention in each trial. As described above, two approaches were employed to mitigate the problem of suboptimal—sparse and inconsistent—information about the interventions. First, qualitative data analysis techniques are used to gather information about interventions: line-by-line coding to ensure that all available information was captured and inductive coding to deal with inconsistencies in the descriptions of intervention features [27]. Second, we employed a broader view of ‘evidence’ than is typical for a systematic review; we examined discussion sections of trial reports to capture trialists’ reflections and accounts of the experience of using the intervention. Although trialists’ accounts cannot be considered *research* evidence, as formal methods were not employed to collect or analyse this data, the use of this *informal* evidence offers much needed insight into the experience of using and delivering an intervention.

We employed these approaches to gather evidence on three dimensions of the intervention:

- The features described as present in electronic prescribing interventions
- The strengths/weaknesses of individual intervention features
- The process of development and implementation

#### Capturing and coding intervention descriptions

With regard to a) capturing evidence on intervention features, we detailed all available information about intervention components as described by the authors. There was a huge variation in the level of detail provided. To provide a crude example, some studies provided descriptions of several hundred words long and provided illustrations, whilst others provided much shorter descriptions; and indeed, one study simply named the intervention as CPOE without providing any description of its characteristics. However, line-by-line coding ensured that we made use of every piece of available information. Inconsistency in the definition and description of features was common; for example, ‘structured order sets’, which allow a prescriber to select with one click a complete order for commonly prescribed medicines, were variously described as ‘order sets’, ‘pre-constructed orders’ and ‘default prescriptions’. Inductive thematic coding enabled us to identify and categorise these similar concepts such that we could ‘map’ the presence or absence of the range of described features to assess how interventions differed from one another.

#### Capturing informal evidence on the experience of developing and using interventions

The inclusion of informal evidence enabled us to go beyond intervention descriptions and examine evidence in the form of real-world experiences of electronic prescribing systems. Few studies employed formal research methods to examine process and implementation issues. However, the vast majority of studies provided a wealth of *informal* evidence via rich description by authors about the perceived strengths and weaknesses of particular features as well as the experience of developing, using and implementing electronic prescribing packages. Such evidence included authors’ reporting of informal feedback from users of the electronic prescribing systems (e.g. hospital doctors), authors’ observations of the impact of electronic prescribing on working practices, and authors’ conclusions regarding associations between intervention features and the success (or otherwise) of the intervention. We employed inductive thematic analysis to produce a narrative structure around the emergent themes.

### Stage two: which intervention characteristics appear to explain differences in outcomes?

In the second stage, to identify which intervention features appeared to be important for successful outcomes, we used the mapped intervention features and the emergent themes from the informal data to re-examine the outcomes of the effectiveness synthesis. We sought to identify whether the small number of studies with negative outcomes were qualitatively different to those with positive outcomes.

### Approaches for mitigating potential weaknesses of the ICA approach

The effectiveness synthesis and ICA were conducted consecutively. However, aside from the primary investigator, they were conducted by different research teams. The independence of the research team conducting the ICA minimised the potential pitfalls of post hoc reasoning.

With regard to capturing evidence from intervention descriptions, it must be recognised that without seeking confirmation from authors about the absence of particular features, it remains unclear whether features that were not described in trial reports were not present in the intervention or whether they were simply overlooked in the description. Confirmation from authors would have increased confidence in the review’s conclusions. However, given the level of informal scrutiny and reflection on the importance of components by authors of the primary studies, it seems reasonable to assume that they would have described and emphasised the features that they considered to have had a discernible impact on outcomes.

Although it provided vital insight into the electronic systems under study, the informal evidence examined as part of the ICA is, of course, at risk of being partial or biased, since formal research methods designed to reduce inherent biases were not employed. We attempted to mitigate this weakness in a number of ways. First, we were explicit about the extent of such data contributing to each theme and the consistency of opinion across the studies. The process of checking that themes that emerged from this data were corroborated by evidence in the effectiveness synthesis in stage 2 of the ICA provided a second validity check. Lastly, following the completion of the analysis, we sought to identify if the themes were corroborated by relevant research identified during the course of the review which did not meet our inclusion criteria, such as qualitative studies.

## Results

### Description of included studies

The review identified 20 trials of electronic prescribing interventions evaluated in paediatric populations [28–47]; the findings showed it to be a largely successful intervention for tackling medication error and other related outcomes in this population although some studies revealed the potential for negative consequences. Fifteen studies examined the impact of electronic prescribing on paediatric medication error (PME), of which nine found statistically significant reductions, a further four found reduced PME although the findings were not statistically significant, and two studies found small non-significant increases in PME. Evidence also suggests that electronic prescribing can reduce mortality rates and adverse drug events (ADE) associated with errors. However, a small number of studies (*n* = 3) suggested that it has the potential to increase harms for children [31, 37, 42]; most notably, one study found a statistically significant increase in mortality following the implementation of electronic prescribing [30]. Full details of the included studies and results of the review can be found in the study report which is available on-line [48].

### Intervention features

The 20 studies all evaluated an intervention for clinicians which involved entering and processing prescription orders via computer as opposed to handwritten paper-based prescriptions. However, there was an array of additional features which varied according to each individual package. The inductive coding process enabled us to map these intervention features at a number of levels. Inductively generated higher level descriptive themes were used to group specific features according to (a) the package type; (b) front-end decision support features; and (c) back-end decision support features.

#### Package types

Analysis of intervention descriptions revealed that electronic prescribing packages varied according to whether they were *unmodified* commercially available packages, *customised* commercially available packages or *bespoke* packages developed by staff in the hospitals in which they were evaluated. Within these types, some packages were designed specifically for use with children whilst others employed generic adult-based systems. As illustrated in Table 2, four studies evaluated unmodified commercially available or ‘off-the-shelf’ packages, of which only one was specifically designed for use with children. Eight studies evaluated commercially available packages that had been customised; in each case, customisations involved making adult packages appropriate for use in a paediatric setting. Six studies evaluated bespoke in-house developed systems; of which all but one were designed specifically for use with children. In two papers, details of the package evaluated were so scant that it was not possible to ascertain whether they were customised or developed specifically for use with children.

**Table 2:** Features of electronic prescribing packages (*n* = 20)

Study (studies with negative findings in italics)	Country	Paediatric specific	Front-end decision support			Back-end decision support		
			Dose calculation	Order sets	Info access	Alerts	Mandatory fields	Access security
Unmodified commercially available packages								
*Han et al. (2005)* [31]	USA					✓	✓	
Jani et al. (2010) [33]	UK				✓	✓	✓	
*King (2003)* [37]	Canada				✓			
Walsh (2008) [46]	USA	✓	✓	✓		✓		✓
Customised commercially available packages								
Cordero (2004) [28]	USA	✓	✓	✓	✓	✓	✓	
Del Beccaro (2006) [30]	USA	✓		✓	✓	✓		✓
Holdsworth (2007) [32]	USA	✓	✓	✓	✓	✓	✓	
Kadmon (2009) [34]	Israel	✓		✓	✓	✓		✓
Kazemi (2011) [35]	Iran	✓	✓	✓	✓	✓	✓	
Keene (2007) [36]	USA	✓		✓	✓			✓
Upperman (2005) [44]	USA	✓	✓	✓	✓	✓	✓	
Warrick (2011) [47]	UK	✓	✓	✓	✓			
Bespoke packages (developed by trialists/hospitals)								
Lehmann (2004) [38]	USA	✓	✓	✓	✓	✓	✓	✓
Lehmann (2006) [39]	USA	✓	✓	✓	✓	✓		
Maat (2013) [40]	Netherlands	✓	✓	✓	✓			
Potts (2004) [41]	USA		✓		✓	✓		
*Sowan (2010)* [42]	USA	✓	✓		✓			
Vardi (2007) [45]	Israel	✓	✓		✓	✓		✓
Unidentified package type								
Barnes (2009) [29]	USA				✓	✓		
Sullins (2012) [43]	USA							

The thematic analysis of informal data on the experience of developing and implementing electronic prescribing revealed a unanimous view among authors that customisation for use with children is critical to the success of interventions (see below). As Table 2 makes clear, two of the three studies with negative findings evaluated systems which were not tailored for use with children specifically. Whilst the third study with negative findings [42] evaluated a paediatric specific tool, it should be noted that this study evaluated *administration* errors rather than prescribing errors. In essence, the study aimed to examine through a simulated exercise the effect of computerised orders, rather than handwritten ones, on nurses ability to detect infusion pump programming errors. As such, the decision support available, whilst paediatric specific, was qualitatively different to decision support for prescribing decisions as examined in other studies.

#### Decision support—‘front-end’ and ‘back-end’ features

‘Front-end’ decision support features of the system [49] are those that are actively accessed and manipulated by the user to support decision-making such as dose calculators, structured order sets and access to other information such as lab results or on-line formularies. System features which were automatically triggered (as opposed to intentionally accessed) were categorised as ‘back-end’ decision support features; they included alerts or warnings about potentially harmful scenarios, mandatory fields (preventing prescribers from continuing with or submitting an order until all necessary fields were completed) or access security such as password entry. Table 2 illustrates which decision support features were described in each study.

The inductive approach, which enabled the higher level categorisation of decision support features, was validated by patterns found in the assessment of informal data on strengths and weaknesses of intervention features. We found that 15 studies commented on the value of ‘front-end’ decision support and were unanimously of the opinion that such features were a key factor in error reduction. By contrast, just four authors commented on the benefits of ‘back-end’ decision support suggesting its relative lack of importance. As can be seen in Table 2, both the Han et al. [31] and King et al. [37] studies had far less sophisticated front-end decision support than many of the other studies (as noted earlier, decision support examined in the Sowan et al. study was not comparable to that evaluated in other studies [42]). Moreover, evidence also supported the relative lack of importance of back-end decision support; despite resulting in increased mortality, the system evaluated in the Han et al. study [31] appeared to have relatively comprehensive ‘back-end’ decision support.

These findings illustrate the value of combining the inductive approach to assessment of intervention descriptions with assessment of informal evidence to provide insight into the strengths and weaknesses of specific components of the intervention. The inductive derivation of features enabled categorisation of inconsistent intervention descriptions and the representation of studies in a tabular format. Alone, this visual juxtaposition of study features would have been sufficient for identification of associations between feature and outcomes. However, the informal data on component strengths and weaknesses provided further confirmatory evidence regarding the validity of the identified associations.

### Developing and implementing electronic prescribing

The studies contained a wealth of informal data regarding the development and implementation of electronic prescribing programmes: five major themes about successful practices emerged. The five themes are described below and summarised in Table 3.

**Table 3:** Example evidence contributing to development and implementation themes

Theme	No. of studies contributing evidence to theme	Informal evidence example	Correspondence between themes and study outcomes
1. Customisation for use with children	14	The risk of failing to customize existing systems to assist with prescribing for pediatric patients is likely substantial.	2 of the 3 studies with negative findings were not customised for use with children. The evaluation in the 3rd study was not designed to test the impact of package type on prescribing.
		(Holdsworth et al. 2007, p. 1064) [32]	
2. Stakeholder engagement	9	Active involvement of our intensive care staff during the design, build, and implementation stages … are prerequisites for a successful implementation.	None of the 3 studies with findings of harm described a stakeholder engagement process.
		(Del Beccaro et al. 2006, p. 294) [30]	
3. Fostering familiarity	13	Probably the most important and fundamental activity necessary for a smooth transition to CPOE is staff CPOE training … Poor training may lead to a lack of system understanding, which can result in frustration, poor acceptance, and a lack of full utilization.	The training provided in the Han et al. study has been identified as inadequate, and no training was described in the other 2 studies with harmful outcomes. Studies measuring at multiple time points show greater benefits at later follow-up.
		(Upperman et al. 2005a, p. e639) [44]	
4. Adequate/appropriate infrastructure	6	Our finding [of an increase in mortality] may reflect a clinical applications program implementation and systems integration issue rather than a CPOE issue per se.	The Han et al. study acknowledges that the harmful outcomes observed were likely due to infrastructure problems rather than EP itself.
		(Han et al. 2005, p. 1511) [31]	
5. Planning and iteration	14	It is important for hospitals to monitor, continually modify, and improve CPOE systems on the basis of data derived from their own institution.	There was a relatively limited (3 months) preparatory phase in the Han et al. study in comparison to other studies.
		(Walsh et al. 2008, p. e427) [46]	

#### Customisation for use with children

A very common theme across the studies was the need to customise generic electronic prescribing systems to render them suitable for use with particular patient groups: 14 studies recommended developing customised electronic prescribing systems or warned against the use of generic ‘off-the-peg’ tools [28, 30–36, 40, 41, 44–47]. Twelve studies specifically indicated the need for paediatric-appropriate tools, for example, with decision support regarding age- and weight-based dosing, the substantial changes in body proportions and composition that accompany growth and development mean that doses of each medicine need to be calculated for each child on an individual basis, rather than being based on a standard dose as for adults. Indeed, three studies emphasised that paediatric-customised systems were an *essential* feature when using electronic prescribing for children [30, 32, 44]. As noted above, when comparing this theme against the findings of the effectiveness synthesis, we found that two studies which found negative findings [31, 37] evaluated off-the-peg commercially available packages not customised for use with children. Indeed, Han and colleagues [31] acknowledged that the harm caused by the electronic system they evaluated may have been due, in part, to the use of a system designed for adult settings.

#### Engaging with stakeholders during development

Nine studies described the involvement of stakeholders in the development of ‘home-grown’ or ‘customised’ electronic prescribing systems; typically, the studies emphasised the benefits of involving multidisciplinary teams or a wide range of different stakeholders for ensuring safety and enhancing relevance and utility. None of the three studies in which harmful outcomes were found described stakeholder engagement.

#### Fostering familiarity prior to implementation

Thirteen of the electronic prescribing studies advocated enhancing familiarity with the electronic prescribing system prior to the ‘go live’ stage. Studies suggested that both acceptability and effectiveness could be detrimentally affected by a lack of familiarity with electronic prescribing systems. This finding was corroborated by the findings of the effectiveness synthesis. The study by Sowan and colleagues [42], which found negative impacts on administration errors, was affected by this factor, noting that relative familiarity with established systems, or perhaps even loyalty to existing systems, has the potential to negate the benefits of a new system. Indeed, Han and colleagues [31] explicitly acknowledged that a lack of familiarity with the system may have contributed to the increased number of deaths in their study. Pre-implementation training delivered in the Han et al. study was described as insufficient by authors of another study who compared their approach to that of Han and colleagues [36]; in the other two studies with negative findings, no such training was described [37, 42]. The strongest evidence, however, came from two studies that examined PME outcomes both immediately following implementation and at later time periods; both found greater benefits at the later time periods [38, 47].

#### Ensuring infrastructure is adequate and appropriate

Six studies commented on the issue of inadequate infrastructure for implementing electronic prescribing [30, 31, 34, 38, 39, 44], for example, the accessibility and availability of electronic prescribing points, the capability of computer systems or the incompatibility of the electronic prescribing system with existing procedures for speeding up the prescription process. Again, Han and colleagues explicitly acknowledged the potential for such infrastructure failures to have contributed to the increased mortalities found in their study: ‘Our finding may reflect a clinical applications program implementation and systems integration issue rather than a CPOE issue per se.’ [31] p. 1511].

#### Planning and iteration

Fourteen studies discussed implementation approaches to support the development of appropriately customised packages; all 14 recommended or implied the value of an iterative approach whereby the electronic prescribing system was customised post-implementation based on user experience and identified needs. However, six of the 14 studies also recommended a long and careful pre-implementation planning phase to pre-empt potential problems. Two studies gave specific details of the length of their pre-implementation preparatory phase, with one study implying that preparation in the Han et al. study was inadequate: ‘The preparatory phase took place during approximately 2 yrs rather than the 3 months described by Han and colleagues’ [36].

In sum, each of the inductively derived themes identified was corroborated in some way by evidence from the effectiveness synthesis. Moreover, the validity of the approach and of employing informal evidence to develop the themes is further underscored as each of the five implementation themes also emerged in an independent qualitative study of the views of UK practitioners on the advantages and disadvantages of electronic prescribing in paediatrics [50].

## Discussion

### Summary of key findings

ICA addresses a critical need for knowledge translation to support policy decisions and evidence implementation. The formal and systematic approach for identifying key intervention content and implementation processes is designed to overcome the deficiencies of poor reporting which often hinders such work whilst also avoiding the need to invest significant amounts of time and resources in following up details with authors—with often uncertain benefits. The inductive approach and analysis of informal data are particularly useful for revealing potentially overlooked aspects of interventions which are actually important for their effectiveness, making it especially appropriate where hypothesised mechanisms in an existing programme theory have failed. A further benefit of the approach is its ability to identify potentially new configurations of components that have not yet been evaluated.

### Strengths and limitations of ICA

Whilst ICA may be an effective method for the development and articulation of hypotheses about critical intervention features, effective approaches for testing such hypotheses would also be desirable. The ICA approach may be a useful precursor to formal analyses of variance in outcomes, such as subgroup analyses and meta-regression. Whilst these analyses may have limited benefit in reviews such as the one in this example (as discussed in the Background section), ICA does offer a formal process through which potentially explanatory theories may be developed, which can then be tested formally using standard statistical techniques.

Qualitative Comparative Analysis (QCA), an approach which has recently been employed in systematic reviews of complex interventions, is another method that may be appropriate for testing the conclusions of an ICA [51, 52]. QCA seeks to identify the necessary and sufficient conditions for an outcome to be obtained and is not subject to the limitations of the statistical methods often used in meta-analysis; it works with small numbers of studies but a large number of possible factors that could explain variation in outcomes and can cope with multiple pathways to success [51, 52]. QCA systematically identifies configurations, or combinations, of various interventions and other contextual characteristics that are (or are not) present when the intervention has been successful (or not) in obtaining a desired outcome and provides metrics for assessing how consistent findings are. This quantification of the link between intervention features and outcomes would enhance the findings of ICA by providing a formal method to validate the generated theories and enable us to move beyond the effective/not effective dichotomy and (though the use of fuzzy sets) rank studies according to the magnitude of their effects. The approach we took is not dissimilar to a ‘crisp set’ QCA without the formal testing for coverage and consistency, which for the small numbers of studies we have in our example, are implicitly assessed in terms of the identification of studies which do not fit a given ‘solution’. Thus, since the QCA method requires features for testing to be identified, the combination of ICA with QCA may be a means to further propel the nascent utility of the QCA method for examining complex interventions, with QCA being particularly useful when the magnitude of effect size estimates needs to be included in the model.

An analysis driven by reviewer interpretation may be susceptible to the biases that a priori specification of data extraction categories in a typical subgroup analysis seeks to prevent. However, its ability to reveal hidden or neglected features, and barriers and facilitators only identified in practical application, means that it is more likely to avoid the pitfalls of simply providing a narrative account describing individual interventions with no real synthesis, which is often the result where there is too much heterogeneity—and too little replication—for meta-analysis to partition the variance successfully. The key strengths of ICA are summarised in Table 4.

**Table 4:** Overall strengths of ICA

• A streamlined approach for producing guidance to support practical application of evidence about effective interventions
• Can uncover potentially neglected aspects of interventions which are actually important for their effectiveness
• Potentially new configurations are identified, which may not actually have been evaluated

### The validity of drawing on informal evidence

Mark Petticrew, in a previous issue of this journal, urged systematic reviewers to ‘re-think’ some of the core principles of systematic review practices in order to develop appropriate evidence in answer to complex questions and about complex interventions [53]. Thus, whilst we recognise the potential dangers of drawing on informal evidence, it could be considered equally imprudent to dismiss outright this underutilised source of experiential data. A wealth of valuable information is often presented in the discussion sections of published reports, and the question for the reviewer is how to understand the merit and utility of this type of knowledge. It is not the outcome of a formal piece of research or process evaluation, certainly, and so cannot be regarded as being equivalent to this form of knowledge. It does however reflect the considered opinion of the authors, in the light of their experiences in conducting their research. Can it therefore be regarded as being similar to the primary data collected as the result of, e.g. interviews and questionnaires? In terms of sampling strategy, there is a clear sampling frame, in that we have the views of a defined set of authors. However, the data may not be as complete as might be achievable through a separate study which sought the views of these participants, as not all may have felt the need to express ‘process’ opinions, and different journals may have different attitudes and requirements for the papers they accept. In addition, the opportunities offered by the rich data available from in-depth interviews clearly go beyond those offered in short sections of a research report. Arguably, the views presented may also be biased, as they may be self-justifying; however, the same weakness can affect data collected through interviews as well, and moreover, these data may actually be more reliable in some ways, as they are the considered and distilled views of the authors, rather than their more instantaneous responses to an interview question. The use of such evidence is perhaps further justified by the lack of a viable alternative; there is a critical need for information with regard to an understanding of the key ingredients of interventions and also a lack of formal process evaluations. As Pawson and colleagues recognise, ‘Information relating to …the subtle contextual conditions that can make interventions float or sink, will be much harder to come by … [reviewers] need to draw judiciously upon a wide range of information from diverse primary sources’ [18]. We therefore regarded this potentially rich and largely ignored source of trialists’ opinions as being a valuable, if potentially incomplete, picture of their experiences with the intervention, treated them as primary data and analysed them accordingly, rather than as the product of robust research for synthesis. We consider the approach to fit with the spirit of evidence-based medicine ‘the conscientious, explicit, and judicious use of *current best evidence* in making decisions’ [54].

### The applicability of the approach

One of the key strengths of ICA is that it works exclusively with information contained within existing trial reports, thereby avoiding the need for the significant additional resources required for author survey or identification of qualitative research. However, despite the acknowledged suboptimal descriptions of the electronic prescribing packages in the synthesised trial reports, some features of paediatric electronic prescribing may have ensured that the nature of information contained within the trial reports was particularly conducive to the ICA approach. First, the inductive approach to coding may have been particularly fruitful due to the newness of electronic prescribing technology. In some ways the newness of the technology can be seen as creating a problem for identifying key intervention features, not least because of inconsistencies in the terminology used. This however, may have also been advantageous for the ICA approach in this instance, since the lack of consensus with regard to terminology meant that authors often provided definition or description as well as naming features. Whilst some intervention descriptions, particularly in later reports, were scant, in other reports, the newness of the technology may have been the reason for the relatively detailed level of description; shorthand descriptions are more likely where there is a well-established intervention, and consensus about what it involves, for example, lengthy descriptions of the features of a parachute (e.g. ‘a folding, hemispherical fabric canopy with cords supporting a harness or straps for allowing a person, object, package, etc., to float down safely through the air from a great height’) are unlikely to be considered necessary since this type of technology has been in common usage for over a century [55]. Moreover, given that many of the interventions were developed in the hospitals in which they were employed and evaluated and that many others were customised, trialists may have been especially motivated to document their developments and innovations with regard to this technology, further enhancing the quality of description. Nevertheless, whilst ICA may thus be less fruitful when there is greater consensus about what an intervention involves (and therefore less extensive description), it must also be recognised that the main aim of the approach is to provide clarity in precisely those situations where an intervention is not well established and there is a lack of consensus about its critical features. A second feature of paediatric electronic prescribing that may have rendered it particularly suitable for ICA was that an early study reported tragic adverse outcomes [30]. It is clear that authors of subsequent trials felt compelled to compare and contrast their findings with this particular trial [30, 32, 36] ensuring a wealth of accounts and rich reflection on the critical differences between successful and non-successful interventions; where less extreme differences in outcomes are observed, it is unlikely that reflection of this sort will occur. However, since this is the first use of the ICA approach and the first time that we are aware of systematic appraisal of this type of informal information, the success of the endeavour in this instance suggests that ICA warrants further application and testing.

### The warrant of the knowledge generated

Whilst it is beyond the scope of this paper to discuss the philosophy of science and evidence-informed decision-making at length, a few observations may be helpful. We have employed an explicitly *inductive* methodology, which means that it is possible that some (or all) of our findings are incorrect and that other factors may actually be responsible for the differences between outcomes observed. Given that most human knowledge is generated inductively though, we do not think that this fact alone should be the cause for rejecting this approach. Indeed, the more traditional statistical subgroup analyses also use induction, so it is clear, we think, that an inductive approach is legitimate. Moreover, had we simply given a narrative account of the studies and their findings, we would have implicitly invited the reader to perform their own informal inductive ICA, and we would argue that our formal inductive analysis is far more systematic and rigorous than any careful reading of intervention summaries might be, as we have systematically sought for key characteristics of studies and their relationships with observed outcomes. Indeed, this approach might better be described as ‘abductive’, ‘retroductive’ or an ‘inference to the best explanation’ form of induction, where all factors, whether convenient (in terms of producing a coherent explanation) or inconvenient, are taken into consideration [56].

Whilst frequently criticised, the formal significance test does provide some degree of protection against the erroneous identification of a relationship between two variables—where in fact, one may not exist; and an ICA does not benefit from this protection (even where we may have taken a difference in magnitude to be meaningful of some real difference between interventions in the ICA). Our response to this critique is threefold. First, we need to acknowledge that this may be true—but we have no way of knowing whether it is or it is not. Second, we observe that we have taken far more ‘variables’ into account in this analysis than would be possible with this small number of studies in a statistical analysis, which actually may give us more confidence in the robustness of our findings. And third, that the warrant gained through the use of inferential statistics—especially with small numbers of heterogeneous studies—may not be all that great, since we are unlikely to have a random sample of all relevant studies. Indeed, we may have all of the relevant studies (a census, where using inferential statistics is obviously illogical), but even if this is not the case, we do not have any evidence to assume we have a genuine probability sample that would lend support to inferences derived from statistical tests. The use of standard statistical methods would have identified significant heterogeneity between studies and, given the fact that they differed in so many different ways, would not have enabled us to go further than to say that we could not explain it and that more research was needed. In the words of Gene Glass, ‘classical statistics seems not able to reproduce the complex cognitive processes that are commonly applied with success by data analysts’ [57].

## Conclusions

Information about the critical features of interventions and guidance for their implementation are essential components of systematic reviews if they are to be more than an academic exercise and the widespread adoption of evidence-based practice more than an aspiration. Intervention Component Analysis is advanced as a promising approach to build up a rich picture of those intervention characteristics which may be associated with successful (and unsuccessful) outcomes. Although the pragmatic nature of ICA means that its findings must be treated with care, it has a useful contribution to make in utilising fully the available evidence.

Since it is the first use of such an approach that we are aware of, there are naturally questions about its validity and applicability. Further methodological work will be essential for testing and refining the process as well as for establishing optimal conditions for its use. Nevertheless, the urgent need to support decision-makers in implementing review findings and the potential utility of the approach as demonstrated within the worked example merit such investigation.

## Abbreviations

ADE: adverse drug events
CONSORT: Consolidated Standards of Reporting Trials
CPOE: Computerised Prescription Order Entry
ICA: Intervention Component Analysis
PME: paediatric medication error
RCTs: randomised controlled trials
